# The Impact of Rheumatoid Arthritis (RA) in Median Nerve Area in the Wrist Joint: A Case-Control Study

**DOI:** 10.7759/cureus.38580

**Published:** 2023-05-05

**Authors:** Abdulmalek Y Abdullah, Rihab A Yousif, Awadia G Suliman, Amel A Ibn Idris, Sujood A Hassan, Shima I Ali, Sultan A Alshoabi, Eman M Algorashi, Bassam N Mohammed, Maisa Elzaki

**Affiliations:** 1 Department of Diagnostic Radiology, University of Medical Sciences and Technology (UMST), Khartoum, SDN; 2 Faculty of Radiology Science and Medical Imaging, Alzaiem Alazhari University, Khartoum, SDN; 3 Department of Diagnostic Radiology Technology, College of Applied Medical Sciences, Taibah University, Al-Madinah Al-Munawarah, SAU; 4 Faculty of Medicine, Gezira University, Khartoum, SDN; 5 Department of Diagnostic Radiology, Sudan University of Science and Technology (SUST), Khartoum, SDN

**Keywords:** ra, high-frequency ultrasound, duration, left median nerve area, right median nerve area

## Abstract

Background

Rheumatoid arthritis (RA) is one cause of carpal tunnel syndromes (CTS); due to increased intracarpal pressure in the rheumatoid wrist, synovial enlargement, joint erosions, and ligamentous laxity cause the compression of the median nerve (MN).

Materials and methods

A case-control study was conducted to assess the measurement of median nerve areas in RA using high-frequency ultrasound (US) and to correlate the measurement with the disease duration. Forty patients with rheumatoid arthritis (RA) and 40 with non-rheumatoid arthritis (RA) as a control group were referred to the radiology department of Yastabshiron Hospital, Khartoum, Sudan, from June to August 2022. After assessing the wrist joint by ultrasound scans, median nerve (MN) cross-sectional area (CSA) measurements were performed using a Fukuda Denshi ultrasound machine (Tokyo, Japan) with a linear-array high-frequency transducer (10 MHz), after receiving ethical approval from the research committee of the faculty of radiological science at University of Medical Sciences and Technology (UMST) and the study participants.

Results

The study demonstrated that the mean measurement of MN cross-sectional area (CSA) in RA patients was 13.60 mm^2 ^for the right and 13.25 mm^2^ for the left MN. The study found that the MN CSA decreased by increasing the disease duration, with significant differences in the median nerve cross-sectional areas in RA and healthy control (p-value of <0.01).

Conclusion

The study concluded that rheumatoid arthritis (RA) had a greater influence on the median nerve cross-sectional areas. MN areas significantly decreased with increasing duration of diseases; the MN cross-sectional areas were more in RA than in the healthy control group.

## Introduction

Rheumatoid arthritis (RA) is a chronic inflammatory autoimmune disorder that primarily affects the body's joints and can affect other organs. RA affects up to 1% of the world's population and majorly affects females leading to disability [1].

A joint working group from the American College of Radiology (ACR) and the European League Against Rheumatism developed in three phases a new approach to classifying RA to refocus attention on the importance of early diagnosis of RA and the institution of effective disease-suppressing therapy to prevent or minimize the occurrence of undesirable sequelae of RA [2]. Carpal tunnel syndrome (CTS) is a known complication of RA that occurs in 30% of patients [3]. The most common finding of CTS in RA patients is synovial tissue inflammation, such as finger flexor tendons tenosynovitis and radiocarpal joint synovitis, rather than median nerve (MN) swelling that occurs in idiopathic CTS [4]. In patients with RA, ultrasonography (USG) measurements of the median nerve can be misleading in diagnosing CTS, and the sonographers should be alert to this topic [5]. The literature found that the combined sensory index (CSI) is CTS's most sensitive electrodiagnostic criterion. A combination of the CSI and the Bland criteria is shown to predict surgical outcomes. USG had high diagnostic accuracy for CTS based on CSI criteria. However, it is unable to distinguish between CSI severity groups [6]. However, other reviews reported that USG is a reliable imaging modality to diagnose CTS [7].

Due to the aforementioned controversy about the effect of RA on the median nerve, this study aimed to investigate the impact of RA on the median nerve measurements at the pisiform level and the subsequent accuracy of USG as the first diagnostic imaging modality to confirm the diagnosis of CTS. This study will be significant to sonographers and radiologists interested in diagnosing RA using medical imaging modalities.

## Materials and methods

A case-control study was conducted in the radiology department of Yastabshiron Hospital, Khartoum, Sudan, from June to August 2022. Forty patients diagnosed with rheumatoid arthritis by rheumatologists and 40 in the healthy control group referred to the ultrasound (US) were included; RA was confirmed by presenting symptoms such as joint pain and swelling and then by the results of multiple blood tests requested by the rheumatologist, such as rheumatoid factor (RF), erythrocyte sedimentation rate (ESR), C-reactive protein (CRP), and complete blood count (CBC). The study's main objective was to assess the impact of rheumatoid arthritis (RA) on the median nerve area measurement at the pisiform level using high-frequency ultrasound; any patient with another disease that affected MN area measurement such as diabetes mellitus and carpal tunnel syndrome (CTS) was excluded. Both wrist joints were scanned by sonographic specialists with experience of more than five years in ultrasound examination, and then, the MN area measurements were performed using a Fukuda Denshi ultrasound machine (Tokyo, Japan) with a high-frequency linear probe of 10 MHz. The measurement of MN was performed in the flexor region. Ethical approval was gained from the research committee of the faculty of radiological science at the University of Medical Sciences and Technology (UMST-FRS 0022/001) and from the study participants.

Ultrasound technique and measurement of MN area

**Figure 1:**
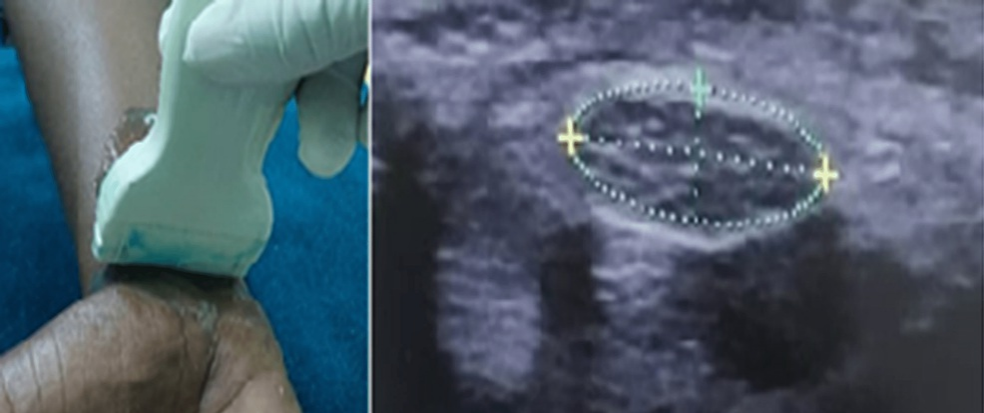
Transverse ultrasound image showing the method of scanning and the measurement of median nerve area in the wrist joint at the carpal tunnel inlet (pisiform level) The photo was taken from the study participants

In this study, the median nerve was examined on both sides of the area of the wrist joint using a high-frequency 10 MHz transducer, with the patient sitting and the forearm resting on support comfortably, the elbow flexed, and the wrist in supination; with proper setting of time gain compensation (TGC) and dynamic range (DR), the image was taken in transverse section at the pisiform level (carpal canal inlet) using flexor pollicis longus tendon as a landmark of a carpal tunnel; then after determining the median nerve and assessing echogenicity, the area was calculated (Figure 1).

Data analysis

The data of this study was analyzed using the Statistical Package for Social Sciences (SPSS) version 23.0 (IBM SPSS Statistics, Armonk, NY). All information gathered via data collection was then coded into variables. Both descriptive and inferential statistics involving the independent t-test, one-way analysis of variance (ANOVA), and Pearson correlation tests were used to present the results. A p-value of less than 0.05 was considered statistically significant.

## Results

This study was conducted in 40 RA patients and 40 healthy non-RA as a control group with the mean age of 45.0±11.86 years and 33.5±9.21 years, height of 170.0±7.00 cm and 170.5±8.21 cm, weight of 75.0±10.54 kg and 75.0±12.18 kg, and body mass index (BMI) of 25.78±4.46 kg/cm^2^ and 25.95±3.68 kg/cm^2^, respectively (Table 1).

**Table 1:** Descriptive statistic for the demographic features of the study participants BMI, body mass index; RA, rheumatoid arthritis; SD, standard deviation

Demographic features	Group	Mean±SD	P-values
Age	RA	45.0±11.86	0.006
	Control group	33.5±9.21	
Weight (kg)	RA	75.0±10.54	0.369
	Control group	75.0±12.18	
Height (cm)	RA	170.0±7.00	0.374
	Control group	170.5±8.21	
BMI (kg/cm^2^)	RA	25.78±4.46	0.854
	Control group	25.95±3.68	

**Figure 2:**
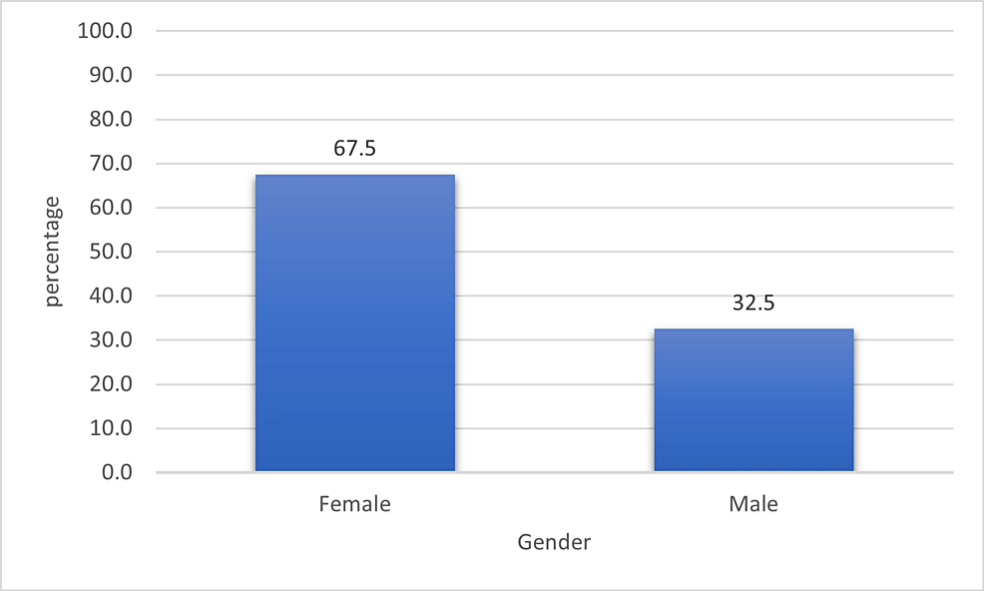
Frequency distribution of RA among genders RA: rheumatoid arthritis

**Figure 3:**
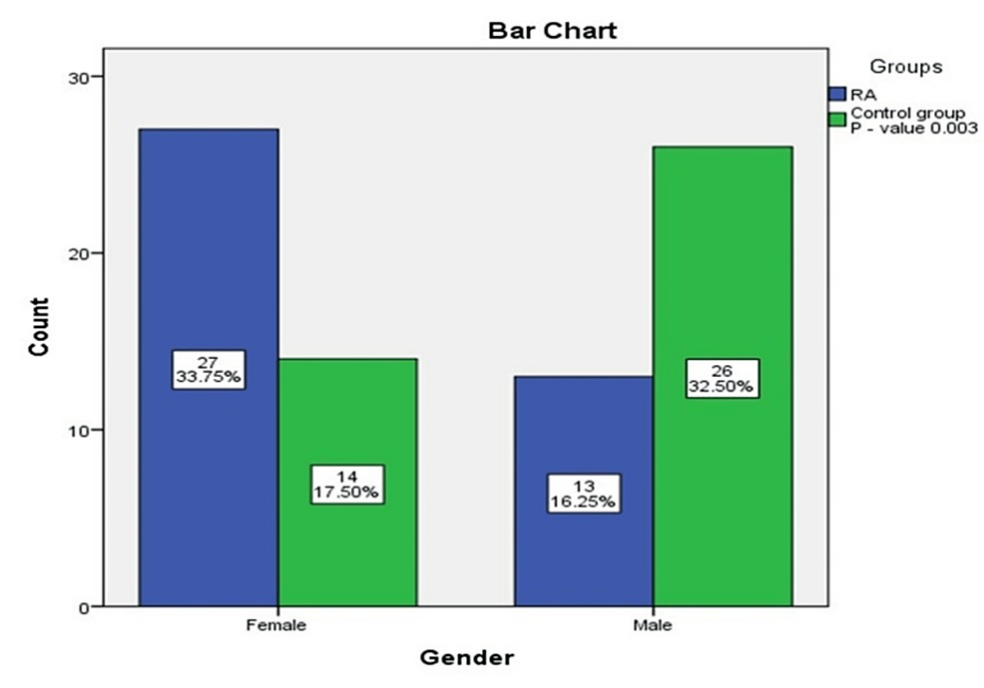
Distribution of gender among the study groups RA: rheumatoid arthritis

RA was more common in females than in males, 67.5% and 32.5%, respectively; the female-to-male ratio was 2.07:1. In the study sampling, there was a good matching of gender among the study groups as there were insignificant differences in gender in both groups (p-value of >0.05) (Figures 2-3).

**Figure 4:**
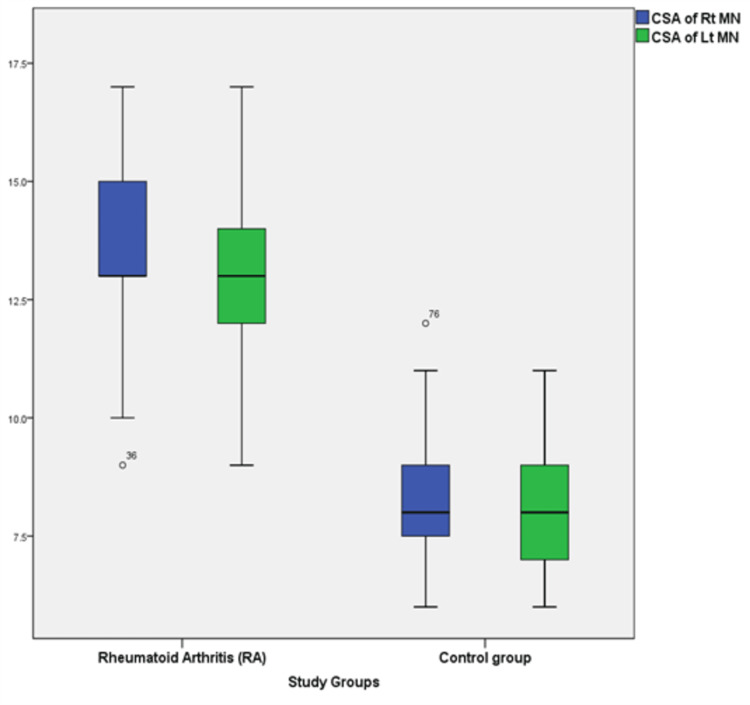
Plot box comparing the mean area of nerve in both study groups (RA and control) CSA, cross-sectional area; Rt, right; Lt, left; MN, median nerve

The study found significant differences in the mean measurement of MN cross-sectional areas (CSA) among the study groups; it was 13.4±1.72 mm^2^ in RA and 8.31±1.30 mm^2^ in the control group, and the mean cross-sectional area in RA patients was 13.6 mm^2^ and 13.25 mm^2^ for right and left MN, respectively, while it was 8.38±1.35 mm^2^ and 8.25±1.28 mm^2^ in the healthy control group (Table 2 and Figure 4).

**Table 2:** Comparison of the mean measurement of median nerve areas in RA and control RA: rheumatoid arthritis

Median nerve CSA (mm^2^)	RA patients	Control group	P-value
	Mean±standard deviation		
Right side	13.6±1.72	8.38±1.35	<0.001
Left side	13.3±1.77	8.25±1.28	
The average for both sides	13.4±1.72	8.31±1.30	

**Figure 5:**
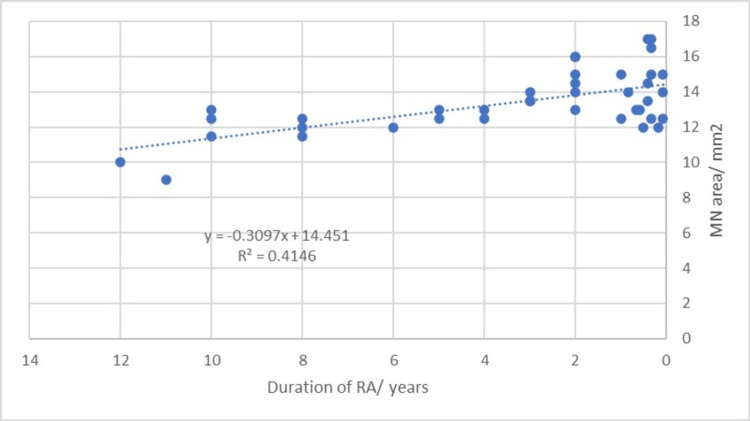
Scatter plot showing an inverse linear association between the duration of RA and MN cross-sectional area measurements RA, Rheumatoid arthritis; MN, median nerve

The study found no significant correlation between age, weight, BMI, and median nerve areas in RA patients (p-values of >0.05, significant positive relation). Between the height of participants and MN, area measurements were noted. A significant inverse relationship was demonstrated between MN area measurements and disease duration (p-value of <0.001) (Table 3 and Figure 5).

**Table 3:** Correlation between age, height, weight, BMI, and duration of RA with MN CSA measurements **Correlation is significant at the 0.01 level (two-tailed). *Correlation is significant at the 0.05 level (two-tailed) BMI, body mass index; Rt, right; Lt, left; MN, median nerve; RA, rheumatoid arthritis; CSA, cross-sectional area

Correlations		Age (years)	Weight (kg)	Height (cm)	BMI (kg/cm^2^)	Duration (years)
Rt MN (mm^2^)	Pearson r	0.189	0.147	0.428^**^	-0.102	-0.619^**^
	Significant (two-tailed)	0.242	0.364	0.006	0.532	0.000
Lt MN (mm^2^)	Pearson r	0.222	0.188	0.345^*^	-0.027	-0.653^**^
	Significant (two-tailed)	0.169	0.245	0.029	0.870	0.000
The average MN (mm^2^)	Pearson r	0.208	0.170	0.390^*^	-0.065	-0.644^**^
	Significant (two-tailed)	0.197	0.294	0.013	0.692	0.000

## Discussion

Nowadays, US is the first diagnostic method used to confirm the diagnosis of CTS and identify the abnormalities in the wrist joint, such as tenosynovitis, ganglion cysts, and joint effusion. The presence of autonomic and entrapped neuropathy often leads to distal sensory or a combination of sensory-motor neuropathy in individuals with rheumatoid arthritis [8]. Carpal tunnel syndrome, which results from the compression of the median nerve beneath the flexor retinaculum in the carpal tunnel, is the most common type of upper limb entrapment neuropathy found in individuals with RA and the general population [8-10]. The structure of the median nerve can be precisely displayed using ultrasonography, and numerous studies have demonstrated that this technique is very sensitive and specific for diagnosing idiopathic CTS [8,11,12].

One study suggests the swelling of the median nerve and the presence of idiopathic carpal tunnel syndrome in patients with rheumatoid arthritis; another study stated that CTS might appear early or be the first indicator of RA [4,13].

In this study, it was found that the measurements of the mean area of the median nerve were more in rheumatoid arthritis individuals than in the control group, and the difference was statistically significant; this result is consistent with Yagci et al. [5] and Onat et al. [14] They observed that the sonographic measurements showed that the measurement of the median nerve CSA in patients with RA was larger than in the healthy controls without clinical and electrophysiological peripheral neuropathy. On the other hand, Hammer et al. [10] and Atan Uzun et al. [15] found no statistically significant differences between patients and controls regarding median nerve CSAs; the study by Hammer et al. [10] reported that 10% of the patients had values that are commonly reported in patients with mild idiopathic CTS; also, Atan Uzun et al. [15] mention that the median nerve CSA was >10 mm^2^ in a higher percentage of RA compared to the control (24% versus 10%); the difference was not deemed statistically significant. In another study by Hammer et al., larger MN CSA was noticed in arthritis patients with CTS than in RA patients and healthy persons without CTS [16].

Additionally, this study revealed a lack of significant correlation between age, weight, BMI, and median nerve CSA measurements in RA patients; however, a significant positive association was observed between the height of participants and MN CSA measurements. Hammer et al. [10] and Atan Uzun et al. [15] found that there were no significant associations between median nerve CSAs and age, height, and weight in RA patients. Another study found that age, BMI, serum uric acid, and microalbumin levels affect RA patients' median nerve cross-sectional area [17].

Moreover, the result of this study is inconsistent with the studies of Hammer et al. [10] and Atan Uzun et al. [15] who stated that an insignificant correlation was noticed between disease duration and CSA measurements of the median nerve; in this study, a significant negative correlation between MN area measurements and disease duration was detected.

This study demonstrates that there was median nerve swelling in rheumatoid arthritis without clinical symptoms of CTS individuals related to the healthy control group.

The main limitation of this study is that it was a monocenter study, so the sample size is insufficient unlike a multicenter study; despite that, this study demonstrated the impact of RA on median nerve. High-frequency ultrasound should be recommended for all patients with RA to aid in the diagnosis and management of the associated condition.

## Conclusions

The study concluded that in rheumatoid arthritis patients, the median nerve CSA at wrist joints was greater than in a control group with a significant difference; the median nerve CSA significantly decreased with the duration of rheumatoid arthritis. There was no significant correlation between age, weight, and CSA of the median nerve in RA patients.
